# Iodine as an Emmanagogue

**Published:** 1856-10

**Authors:** 

**Affiliations:** Columbia, Illinois


*Iodine as an Emmenagogue.* By DR. SHOEMAKER, of Columbia, Illinois.

I am just "Old Fogy" enough, to be surprised that Young Physic should give us another emmenagogue to add to the long-tailed list left us by the venerable Chapman, and many other well learned men of by-gone days.

Some one (I think it was W. P. Dewees) has defined menstruation as "a periodic discharge from the uterus of a colored fluid resembling blood, liable to be interrupted by *pregnancy*, *suckling* and *disease!*" I, with many others, wiser than myself, accept this definition and act accordingly —that is, let the subject alone during the two first and natural causes of arrest, and try to remedy the last, let it be what it may. A *supposed* "rheumatism of the uterus," as suggested by some—then, if we are not on the hobby of Alkali and Acid, as being, in turn, cause and cure—perhaps we might give some of Dewees' Vol. tinct. of Guiac, but not Iodine! Is the patient rather an imprudent one, who, about the time the flux should appear—in good health and full habit, with skin soft and perspiring, intentionally or otherwise gets her feet wet, or from some cause takes cold—amenorrhœa or dysmenorrhœa is the consequence, headache, injected eyes, &c. We give her some warm tea, sit over a hip-bath, and put the legs to the knees in a mustard bath: three fluxes ensue, one from the nose, one from the skin, and one from the uterus—she is well! but has not taken or required any Iodine!

Old Fogies do not believe that typhus fever is *caused* by an ulcerated condition of the abdominal glands, but *vice versa*—just so amenorrhœa and dysmenorrhœa are effects, not causes. Young Physic may expect to relive many patients, whose health is bad, and who suffer from suppressed or painful menstruation, by iodine, or any other of the so-called emmenagogues. We must seek to restore the general health by remedies indicated by the symptoms present; succeed in this, and the amenorrhœa will be cured. It is true, however, that cases may and often do occur that iodine is the *remedy indicated!*

Cullen and most others who diligently searched for what they thought *should* exist, viz: a true emmenagogue, finally gave it up in their minds, but really found it in a pair of flannel drawers with a waistband that covered the whole pelvis—in rusty iron, that improved the blood, when aided by exercise, beef-steak, and hilarity! But iodine is good, too, *in some cases.*

Dysmenorrhœa is often caused by one of the hyperæmic conditions of the uterus—say determination to the part retarded in its transit, (congestion,) or by determination; the circulation in the part, partly increased, and partly diminished (inflammation); in these cases, what are the indications—iodine, or the lancet and anodines?—*St. Louis Med. & Surg. Journal.*

